# “What Kind of Nurse Will I Be?”: a qualitative descriptive study of nursing students’ experiences in obstetric and gynaecologic clinical practice

**DOI:** 10.1186/s12912-026-05160-3

**Published:** 2026-08-08

**Authors:** Gülay Akman, Gamze Yavaş

**Affiliations:** 1 Department of Obstetrics and Gynecology Nursing, Faculty of Health Sciences, Ondokuz Mayıs University, Kurupelit Campus, Atakum, Samsun, 55139 Turkey; 2 Department of Obstetrics and Gynecology Nursing, Kumluca Faculty of Health Sciences, Akdeniz University, Antalya, Turkey

**Keywords:** Clinical practice, Emotional support, Nursing students, Obstetrics and gynaecologic, Professional identity, Self-efficacy

## Abstract

**Background:**

Obstetric and gynaecologic clinical practice may expose nursing students to emotionally demanding situations and complex care interactions that shape emerging professional identity and confidence.

**Aim:**

The aim of this study was to explore nursing students’ care experiences during their obstetric and gynaecologic clinical practice.

**Methods:**

A qualitative descriptive design was used. All eligible third-year nursing students who had participated in obstetric and gynaecologic clinical practice for the first time were recruited for the study. Data were collected through written reflective care stories at the end of the rotation. Narratives from 35 students were analysed using qualitative content analysis, following Graneheim and Lundman.

**Results:**

Four main themes emerged from the students’ narratives: (1) Professional identity questioning and humanistic care orientation, (2) Self-discovery and understanding nursing beyond tasks, (3) Struggles and barriers in providing emotional support, and (4) Satisfaction with the care offered and perceived self-efficacy. Students described reflecting on the kind of nurse they aspired to become, recognizing the importance of emotional presence alongside technical care, experiencing uncertainty in highly sensitive situations and reporting satisfaction with the care they offered and a sense of perceived self-efficacy when they addressed patient needs through education, problem-solving, and supportive presence.

**Conclusions/implications for practice:**

Clinical learning environments and role modelling may influence students’ professional socialization in obstetrics and gynaecologic settings. The findings may inform educational approaches involving communication skills training, supervised reflective learning, and guidance for emotionally challenging encounters, which may support students’ perceived confidence in providing compassionate, person-centred and holistic care.

**Supplementary Information:**

The online version contains supplementary material available at https://doi.org/10.1186/s12912-026-05160-3.

## Introduction

Considering that nursing is a practice-based profession, clinical education and practice constitute a significant portion of nursing education, and theoretical education serves as a complementary component to clinical instruction and practice [1]. The goals of nursing education are to help nursing students reconstruct their nursing identity, develop a care vision, and to gain and internalize critical thinking and problem-solving skills [2, 3]. These can only be achieved through clinical practice, creating a complex and dynamic learning environment [4]. Clinical practice is an integral and fundamental component of nursing education [5]. Clinical practice provides nursing students with opportunities to integrate their theoretical knowledge with cognitive, affective, and psychomotor skills, playing a central role in bridging the gap between theoretical learning and real-world nursing experiences [6, 7].

In Turkey, nursing education is designed to meet national and international standards, following the National Qualifications Framework for Higher Education in Turkey [8] and the National Nursing Core Curriculum [9]. Undergraduate nursing programs in Turkey span at least four years, encompassing a minimum of 4600 h of education, with approximately half allocated to clinical practice and one-third to theoretical coursework [10]. The courses with clinical practice in the nursing curriculum in Turkey are Fundamentals of Nursing, Medical-Surgical Nursing, Obstetrics, Women’s Health and Diseases Nursing, Pediatrics Nursing, Nursing Management, Nursing Education/Instruction, Psychiatric Nursing, and Public Health Nursing. All nursing education courses with clinical practice allow students to develop essential skills and professional identities [11, 12]. Clinical practice in Turkey thus serves as a cornerstone of nursing education, providing students with opportunities to experience diverse healthcare settings and to translate theoretical knowledge into practical skills.

Nursing students are exposed to a variety of practice settings throughout their education. Clinical placements play a significant role not only in acquiring professional knowledge and skills but also in fostering students’ competence and awareness of professional identity and values [13, 14]. However, students may face various challenges when encountering new or unfamiliar clinical environments [15, 16]. In this context, understanding students’ experiences, emotional reactions, and challenges encountered, as well as how they cope with these challenges during clinical placements, is critically important for their personal and professional development [17, 18]. There are numerous studies in the literature that have examined the experiences of nursing students in various clinical settings such as burn units [19], psychiatric clinics [20], pediatric clinics [21], operating rooms [22], and emergency and intensive care units [23]. Previous studies have also examined nursing students’ experiences in maternal-newborn or obstetric contexts, including pre-rotation concerns, anxiety, breastfeeding support expectations, and broader lived experiences in a mother-and-child centre [24–26]. However, less is known about how obstetric and gynaecologic clinical placements contribute to students’ professional identity formation, particularly in relation to their responses to emotionally challenging encounters such as perinatal loss and adolescent pregnancy, as well as the factors that shape the development of compassionate, person-centred care in this context. This underexplored area is important because obstetric and gynaecologic settings involve high emotional intensity alongside intimate and gender-sensitive forms of care, constituting a distinct clinical learning environment.

For nursing students, encountering and providing care for a pregnant woman, a new mother, or a newborn baby for the first time during their obstetric and gynaecologic clinical placements can be a stressful experience [25]. Therefore, gaining an in-depth understanding of nursing students’ experiences during obstetric and gynaecologic clinical placements is crucial for enhancing clinical education, informing educators and clinical nurses about students’ learning and support needs and supporting the development of appropriate care behaviours to meet the holistic needs of women.

In this context, this study was informed by a professional socialisation perspective. Professional socialisation conceptualises professional identity formation as an ongoing process through which nursing students internalise professional values, norms, role expectations, and ways of relating to patients through education, clinical practice, and interaction with role models [27, 28]. From this perspective, clinical placements are not only settings in which students practise technical skills, but also social learning environments in which they observe professional behaviours, negotiate their own values, and make sense of the kind of nurse they wish to become. This perspective is particularly relevant to obstetric and gynaecologic clinical practice because students encounter emotionally demanding, intimate, and gender-sensitive forms of care that may make professional identity work, humanistic care orientation, and emotional preparedness especially visible.

Accordingly, this study aimed to explore nursing students’ care experiences during obstetric and gynaecologic clinical practice.

## Methods

### Design and setting

In this study, a qualitative descriptive design was used. This approach was selected to provide a straightforward, naturalistic description of the phenomenon by summarizing participants’ specific experiences in everyday terms and staying close to the data to capture the who, what, where, and why of these events, generating practical insights for nursing education and clinical practice [29–31]. The reporting of this study adhered to the Standards for Reporting Qualitative Research (SRQR) [32] guidelines.

The study was conducted at a university in northern Turkey, Faculty of Health Sciences, Department of Nursing, during the clinical practice rotation of the obstetrics and gynaecologic nursing course in the fall semester of the 2021–2022 academic year. The clinical practice for this course consisted of students attending two days a week, eight hours a day, for a total of 10 weeks, following the first four weeks of theoretical and laboratory instruction. Clinical placements were conducted at the same university’s Health Practice and Research Center, specifically in the obstetrics and gynaecologic units, the maternity ward, and the outpatient clinics.

### Participants

The study population consisted of 135 third-year nursing students enrolled in the obstetric and gynaecologic nursing course’s clinical practice during the fall semester of the 2021–2022 academic year at a university in northern Turkey. All eligible students were invited to participate. Students who met the inclusion criteria (1) enrollment in the course, (2) completion of the clinical placement, and (3) willingness to submit a written reflective care story and who voluntarily provided written narratives constituted the study sample. A total of 35 students submitted reflective care stories, which were included in the analysis. The final sample comprised 33 female and 2 male students, aged 20–22 years. The voluntary nature of participation may have introduced self-selection bias, as students who contributed narratives may have differed from those who did not in terms of motivation, engagement with clinical learning, or willingness to engage in reflective writing. As a result, the experiences of students who were less inclined to participate or who had more neutral clinical experiences may be underrepresented in the dataset.

### Data collection

Data were collected through students’ written reflective care stories (narratives). All eligible third-year students enrolled in the obstetric and gynaecologic nursing clinical practice were invited to participate, and those who volunteered provided a written care story. Students completed a 10-week clinical rotation and on the final day, they wrote a narrative describing the most impactful real-life care experience they encountered during the obstetric and gynaecologic clinical practice and reflected on their thoughts, emotions, clinical observations and nursing actions related to that event. In qualitative descriptive research, written narratives and personal documents may be used to capture participants’ firsthand accounts of the phenomenon in their own words [29, 31].

To support and structure reflective writing, students received a Reflective Care Story Writing Guide developed by the researchers for this study (Supplementary File 1). The guide was developed iteratively: draft prompts were initially constructed based on a review of the reflective writing and qualitative nursing education literature, then reviewed for content validity by two nurse academics with experience in qualitative research who were external to the study team. Revisions were made following their feedback, with particular attention to ensuring the prompts were open-ended and did not channel responses toward predetermined themes. The revised guide was piloted informally with three nursing students from a previous cohort (not enrolled in the study) to assess clarity and feasibility within the 40–45 min writing session; minor wording adjustments were made based on their feedback before final use. The guide included open-ended prompts covering five areas: (1) the specific care experience they witnessed and participated in, (2) their internal emotional and cognitive responses, (3) their clinical observations and nursing actions, (4) the overall impact of the experience on their perspective regarding patient care, and (5) their professional self-discovery. Students also provided brief demographic information (age and gender) on the first page of the writing guide. To minimize potential power dynamics, the writing session was supervised by an independent faculty member who was not involved in teaching or evaluating the students. Before the writing session, the independent faculty member received a brief orientation and provided a standardized explanation regarding the study aim, voluntariness, confidentiality, and instructions for completing the care story. Students wrote their care stories individually in a quiet classroom within the hospital; the writing session lasted approximately 40–45 min. Students were assured that there were no right or wrong answers, emphasizing the value of their authentic subjective experiences. All participating students submitted their handwritten care stories. The reflective care stories were written by students in Turkish, their native language, to ensure authentic and uninhibited expression. The handwritten care stories were typed into Microsoft Word and de-identified. Narratives selected for quotation in the manuscript were translated into English by the researchers, both of whom are proficient in English, and reviewed by a bilingual nurse academic external to the study team to ensure semantic accuracy and conceptual equivalence. The translated quotations were then back-translated into Turkish by an independent bilingual researcher to verify fidelity to the original meaning before final inclusion.

### Data analysis

Data analysis was performed using the qualitative content analysis approach described by Graneheim and Lundman [33]. This approach was selected as it is commonly used in qualitative descriptive studies and supports analysis that stays close to participants’ accounts and emphasizes naturalistic descriptions rather than highly abstract interpretations [29, 30]. Both researchers independently contributed to all stages of analysis. First, the care stories were read repeatedly to achieve immersion and gain an overall understanding of the dataset. Next, the texts were segmented into meaning units relevant to the study aim. Meaning units were then condensed while preserving their core meaning and assigned codes. Codes were compared for similarities and differences and grouped into categories. Finally, categories were reviewed, refined, and abstracted into overarching themes.

To enhance the trustworthiness of the findings, an audit trail was maintained throughout the analytic process. The researchers independently coded the narratives and subsequently compared their coding decisions. Any discrepancies were resolved through discussion, with narrative extracts re-examined in their full context until consensus was reached. No formal inter-rater reliability statistic was calculated, consistent with the epistemological stance of qualitative content analysis, which prioritizes interpretive consensus over numerical agreement indices. Peer debriefing sessions with an external nursing faculty member provided an additional check on the coherence and plausibility of emerging codes, categories, and themes. The coding framework underwent three iterative revision cycles, during which categories were refined, merged where appropriate, and re-examined against the full dataset to ensure coherence and consistency with participants’ narratives.

Regarding data adequacy, saturation was not used as a criterion for stopping data collection because the study relied on a fixed dataset of voluntarily submitted narratives rather than ongoing recruitment. Instead, thematic sufficiency—the point at which further analysis yielded no new categories or substantial conceptual insights—was used as the analytic criterion. The research team determined thematic sufficiency during the third coding cycle, when further review of the narratives produced no new categories, existing categories were repeatedly supported by multiple meaning units, divergent and ambivalent narratives could be accommodated within the existing thematic structure without altering its conceptual boundaries, and both researchers reached consensus that the dataset provided sufficient analytic depth for the qualitative descriptive aim.

To further support the credibility of the findings, member checking was conducted. The preliminary thematic structure was sent to all participating students, and participation in this process was entirely voluntary. Eight participants (23% of the sample) provided feedback, indicating that the themes were broadly recognizable and reflective of their experiences. No respondents suggested changes to the coding structure or thematic interpretations; therefore, no revisions to the thematic framework were required. However, the relatively low response rate means that member checking should be interpreted as limited participant feedback rather than comprehensive validation of the findings.

Systematic attention was also given to divergent cases throughout the analytic process. Narratives that appeared to complicate or challenge emerging interpretations, including accounts describing more neutral or ambivalent experiences, were discussed by both researchers and incorporated into the final analysis. No narratives were excluded, and contradictory or less typical accounts were retained to support a more nuanced interpretation of the data.

### Rigor and trustworthiness

To enhance the rigor and trustworthiness of this study, strategies addressing credibility, dependability, confirmability, and transferability were employed. Dependability was supported by providing detailed descriptions of the study procedures and analytic processes. Credibility was strengthened through independent data analysis by two researchers and the use of verbatim quotations to demonstrate clear links between participants’ accounts and the findings. As noted in the Data Collection section, an independent faculty member supervised data collection to minimize power dynamics and social desirability bias. Confirmability was supported through peer debriefing with an external nursing faculty member experienced in qualitative research who reviewed the coding structure and thematic map. Transferability was facilitated by providing a detailed description of the study sample and the clinical context.

### Reflexivity

Reflexivity was considered throughout the research process. Both researchers are nurse academics with experience in nursing education and qualitative research. One researcher was employed within the same faculty where participants were enrolled, whereas the other was affiliated with a different institution. Neither researcher held a direct teaching or evaluative role in the obstetric and gynaecologic nursing course from which participants were recruited. Specifically, both researchers hold a humanistic orientation toward person-centred, compassionate care, which may have predisposed them to attend more closely to narratives that reflected these values during coding and theme development. These preconceptions were actively discussed before and during analysis, and reflexive memos were maintained throughout to monitor their potential influence on interpretive decisions. Prior to analysis, the researchers discussed their professional backgrounds, assumptions, and expectations regarding students’ clinical learning experiences in obstetric and gynaecologic settings. Reflexive notes were maintained throughout coding and theme development to enhance awareness of potential preconceptions and to critically examine how familiarity with the educational and clinical context might shape data interpretation. Although the researchers had no direct role in grading or evaluating participants, and an independent faculty member supervised the writing sessions to reduce potential power imbalances and social desirability influences, it remains possible that students were aware of the researchers’ institutional roles. This awareness may have influenced the content or tone of their narratives, a possibility that was considered throughout the analytic process.

### Ethical considerations

Ethical approval for the study was obtained from the Social and Human Sciences Research Ethics Committee of Ondokuz Mayıs University (Decision number: 2021/169, Decision date: 26.02.2021). The study was conducted in accordance with the principles of the Declaration of Helsinki and relevant publication ethics. Prior to data collection, written and verbal informed consent was obtained from all participating students. The reflective care story was not a mandatory course assignment; it was an optional research activity conducted at the end of the clinical rotation. Students were explicitly informed that choosing not to participate would have no bearing on their clinical grades or course standing. The students were assured that their participation in the research would not affect their performance assessment in clinical practice. To protect participant privacy and maintain data confidentiality, anonymous codes (S1-S35) were used instead of their names throughout the data analysis and reporting processes.

## Results

The qualitative content analysis of the written reflective care stories revealed four main themes depicting the students’ clinical experiences (See Table 1).

**Table 1:** Themes, subthemes/categories, illustrative codes, and representative quotations

Themes	Subtheme /Category	Illustrative codes	Representative quotations
Theme 1. Professional identity questioning and humanistic care orientation	Questioning nursing as a career	Uncertainty about becoming a nurse; questioning nursing as the right profession; doubting career choice	*“I have never been sure that I would work as a nurse. Have I really wanted to work in nursing?” (S15)*
	Defining the nurse I want to be/want to avoid	Defining the nurse I want/avoid; setting professional boundaries; non-judgmental stance	*“I don’t know what type of nurse I will become*,* but I know what type of nurse I don’t want to become… my first goal is to be a helpful person who does not underestimate or judge what people experience.” (S13)*
	Commitment to humanistic, non-judgmental care	Made a promise to myself; care without judgment; touching lives; with understanding	*“I promised myself that I would practice nursing as I was taught—with understanding—trying not to upset or hurt anyone*,* and most importantly*,* without judging people*,* but by touching their lives.” (S1)*
	Learning through role modelling	Task-focused care; harsh tone; need for explanation before procedures; respectful communication	*“The nurse may be right; it might be difficult for her*,* but when she entered the patient’s room*,* she should have introduced herself*,* explained the procedure she would perform and why*,* and informed us what would happen after the procedure. If I had been a nurse*,* I would have behaved as I described before.” (S20)*
Theme 2. Self-discovery and understanding nursing beyond tasks	Self-discovery: sense of belonging and self-awareness	Feeling of belonging; unit as a place I feel happiest; learning about myself	*“I think that the obstetric and gynecologic nursing unit is a place that I like and belong to most and where I feel the happiest.” (S22)*
	Self-discovery: reflecting on task-focused care and the need to inform patients	Realising being task-focused; explaining procedures and results, improving communication	*“At the beginning of the clinical practice*,* I realized that I was task-focused since I was performing many tasks for the first time. Later*,* when the woman asked why the fetal monitoring was being repeated*,* I explained that it was part of the routine. This taught me that I must inform patients about why I perform every procedure and their results.” (S33)*
	Discovering the profession: understanding nursing beyond tasks	Listening carefully; understanding patients; support as ‘more effective than medication’	*“…I discovered that a nurse must carefully listen to patients and understand what they say.” (S30)*
	Discovering the profession: reframing the meaning of care	Respectful treatment; how care is provided is as important as what is provided	*“I figured out that how nurses provide care and treat patients is as important as what care they offer. However good we are at nursing*,* the care we give loses its importance when we do not treat patients well or respect them.” (S29)*
Theme 3. Struggles and barriers in providing emotional support	Communication barriers in emotionally intense situations; being at a loss for words	Could not say a word; not knowing what to say; wanting to allow sharing of sadness	*“The patient had a planned pregnancy at the age of 26 years and was very excited. After she had an examination and ultrasound*,* the doctor told her that she had experienced a miscarriage… I couldn’t say a word…I wish I had been able to talk to her and allowed her to share her sadness.” (S12)*
	Freezing/hesitation in challenging encounters; feeling helpless in the face of distressing scenes	Staring; freezing for a while; not knowing what to do; uncertainty how to respond	*“The nurse spoke in a harsh tone during the bleeding check and prevented the patient from holding onto her arm for support… The nurse said*,* ‘You need to cooperate*,*’ and left the room. I just stared at the nurse; even after she left*,* I kept thinking about how she treated the patient. I froze for a while.” (S35)*
Theme 4. Satisfaction with the care offered and perceived self-efficacy	Professional satisfaction from helping and problem-solving	Doing something useful; solving problems; relieving discomfort; professional fulfillment	*“Showing interest in a baby*,* finding solutions to its problems*,* relieving its discomforts*,* and knowing that we did something useful created a very special feeling.” (S16)*
	Perceived self-efficacy	Perceived confidence in providing education and support; feeling useful; perceived ability to contribute to care	*“I was giving education to the mother and her relatives about breastfeeding and helping the mother to breastfeed her baby …I felt as if I had been Pollyanna.” (S21)*

### Theme 1: Professional identity questioning and humanistic care orientation

During their clinical practice, students questioned their professional identity and whether nursing was the right occupation for them:

*I have never been sure that I would work as a nurse. Have I really wanted to work in nursing? (S15*,* female)*

Students also articulated the kind of nurse they aspired to become:

*I promised myself that I would practice nursing as I was taught—with understanding—trying not to upset or hurt anyone*,* and most importantly*,* without judging people*,* but by touching their lives. (S1*,* female)*

Some students described defining the kind of nurse they wanted to be or avoid based on what they observed in practice:

*The nurse was going to administer medication through the vagina. Mrs. S. could not cooperate because she was scared. The nurse may be right; it might be difficult for her*,* but when she entered the patient’s room*,* she should have introduced herself*,* explained the procedure she would perform and why*,* and informed us what would happen after the procedure. If I had been a nurse*,* I would have behaved as I described before (S20*,* female)*

*I don’t know what type of nurse I will become*,* but I know what type of nurse I don’t want to become… my first goal is to be a helpful person who does not underestimate or judge what people experience. (S13*,* female)*

Students described witnessing care that appeared task-focused and less responsive to patients’ emotional needs:

*Nurses in the ward do not care much about how patients feel; they just perform their tasks. They did not try to talk to or comfort Mrs. S. Instead*,* they did nothing to comfort her. When I saw how Mrs. S. was being treated*,* I felt sad. I empathized with her. If I had been Mrs. S.*,* I would have wanted someone to talk to me*,* comfort me*,* and give me information about the procedure. (S20*,* female)*

Students described valuing emotional presence and compassionate behaviors in addition to technical care:

*Creating a positive influence on the lives of people leads to the feeling of happiness (S3*,* female)*

*She hugged me and said*,* “You will become a very tenderhearted nurse”. That day*,* I promised: “I will become a person and care centered nurse*,* not a task focused one. (S33*,* female)*

*Mrs. S. started to cry. I patted her shoulder and told her that everything was under control and that I would stand by her. I persuaded her not to get anxious. (S2*,* female)*

*She was just 17 years old and she was scared. I wanted her to feel that I would support her; I touched her shoulder and said ‘there won’t be any problems by smiling. (S13*,* female)*

### Theme 2: Self-discovery and understanding nursing beyond tasks

During their clinical practice, students described a process of exploration and meaning-making that increased self-awareness and clarified their understanding of nursing beyond technical tasks. While Theme 1 captures students’ evaluative and moral reflections triggered by observing others—particularly their questioning of task-focused care and their articulation of the nurse they aspired to become—Theme 2 documents an inward, experiential process of personal growth and professional discovery arising from students’ own participation in care. The conceptual distinction is thus between externally prompted identity questioning (Theme 1) and internally generated self-discovery through active engagement (Theme 2).

### Subtheme: Self-discovery

Students described the clinical practice of obstetric and gynaecologic nursing as an opportunity for personal exploration, including a sense of belonging and reflection on their own emerging approach to care.

*I think that the obstetric and* gynaecologic *nursing unit is a place that I like and belong to most and where I feel the happiest (S22*,* female)*

*I’d like to learn everything since I’m a woman and I want to know everything about myself. (S21*,* female)*

After initially focusing on procedures, one student described learning to explain the purpose and implications of routine clinical monitoring to patients:

*At the beginning of the clinical practice*,* I realized that I was task-focused since I was performing many tasks for the first time. Later*,* when the woman asked why the fetal monitoring was being repeated*,* I explained that it was part of the routine. This taught me that I must inform patients about why I perform every procedure and their results. (S33*,* female)*

### Subtheme: Discovering the profession

Students described gaining a clearer understanding of nursing beyond technical tasks, highlighting listening, supportive presence and respectful communication as central to care.

*I couldn’t imagine that Mrs. F. could change my view of nursing. (S33*,* female)*

*…I discovered that a nurse must carefully listen to patients and understand what they say. (S30*,* female)*

*…I found that there is something better than medications given by nurses: that is*,* provision of support. (S2*,* female)*

*I figured out that how nurses provide care and treat patients is as important as what care they offer. However good we are at nursing*,* the care we give loses its importance when we do not treat patients well or respect them. (S29*,* female)*.

### Theme 3: Struggles and barriers in providing emotional support

Students described difficulties providing emotional support in situations involving vulnerability, grief, or distress, including uncertainty about what to say or how to respond. For instance, encountering sudden pregnancy loss left students at a loss for words:

*The patient had a planned pregnancy at the age of 26 years and was very excited. After she had an examination and ultrasound*,* the doctor told her that she had experienced a miscarriage… I couldn’t say a word…I wish I had been able to talk to her and allowed her to share her sadness. (S12*,* female)*

Students also described freezing in the moment when witnessing harsh, task-focused interactions:

*The nurse spoke in a harsh tone during the bleeding check and prevented the patient from holding onto her arm for support. […] The nurse said*,* ‘You need to cooperate*,*’ and left the room. I just stared at the nurse; even after she left*,* I kept thinking about how she treated the patient. I froze for a while. (S35*,* female)*

In addition, limited experience with specific vulnerable groups, such as adolescent pregnancies, created communication barriers and hesitation:

*I met a 16-year-old pregnant woman. To be frank*,* I hesitated to give care to her since I didn’t know how to start communication*,* what to tell her*,* and how she would react. (S32*,* female)*

### Theme 4: Satisfaction with the care offered and perceived self-efficacy

Students described satisfaction and subjectively reported confidence when they perceived that their nursing actions were helpful and responsive to patients’ needs. They linked these feelings to solving problems, providing education, and being emotionally present during care.

*Showing interest in a baby*,* finding solutions to its problems*,* relieving its discomforts*,* and knowing that we did something useful created a very special feeling. (S16*,* female)*

*I was giving education to the mother and her relatives about breastfeeding and helping the mother to breastfeed her baby …I felt as if I had been Pollyanna (a famously optimistic fictional character). (S21*,* female)*

Some students also described positive feelings when witnessing patients’ and families’ joy and meaningful moments in care:

*[…] Witnessing joy experienced by people and that blessed moment when the mother and her baby met has made me happy. (S15*,* female)*

Others emphasized satisfaction when practical problem-solving was combined with supportive presence:

*Influencing the life of a person*,* creating solutions to her problems and most importantly holding her hands and saying ‘I’m here with you’*,* and knowing that I did something very useful create a very precious feeling. (S27*,* female)*

## Discussion

This study explored nursing students’ experiences during obstetric and gynaecologic clinical practice through written reflective care stories. Reflective writing can be a valuable approach for gaining insight into how nursing students describe their emerging professional identity and perceived competence. Previous studies have highlighted reflective writing as a method for expressing emotions and supporting personal development during the process of becoming a nurse [12, 17, 34]. Recent studies and reviews suggest that reflective writing and structured reflection prompts may support nursing students in processing clinical experiences and linking these experiences to perceived learning and professional growth during placements [35–37]. Supporting students in articulating and reflecting on their clinical experiences may help educators identify learning needs that extend beyond technical skills.

Consistent with our first theme, students commonly questioned their professional identity and whether nursing was the right occupation for them. These reflections were shaped by the clinical environment, particularly by observing practicing nurses during routine care. Witnessing care that appeared predominantly task-focused and less responsive to patients’ emotional needs prompted students to reflect on their own values and to articulate the kind of nurse they aspired to become. Their accounts indicated a growing orientation toward humanistic, person-centred care, emphasizing the importance of emotional presence alongside technical and curative tasks. Similar to our findings, González-García et al. [23] reported that students decided on the type of nurse they wanted to be based on their observations during clinical placements. Consistent with this finding, Upasheva et al. [26] identified “transformative learning: from observation to aspiration” as a major theme among nursing students in a mother-and-child centre, suggesting that clinical observations may shape students’ professional aspirations and their emerging perceptions of the kind of nurse they wish to become. Saarikoski et al. [38] emphasized that role modelling is fundamental to professional identity development, while Baldwin et al. [39] and Donaldson [40] highlighted that students may encounter both positive and negative role models, with “good” role models supporting confidence and competence. Recent studies similarly show that students attend closely to clinicians’ professional behaviours as role models [41] and that internship-based modelling contributes to professional identity development [42]. Longitudinal evidence further suggests that identity formation follows different pathways shaped by the interplay between personal values and clinical experiences [43]. Together, these findings reinforce the clinical environment as a site of value formation and identity development. In our study, encountering task-oriented care appeared to prompt internal questioning and a clearer articulation of humanistic values, highlighting the need for clinical educators to promote positive, humanistic role modelling. In this context, students’ emphasis on explaining procedures before initiating care (e.g., S20) also reflects respect for patient autonomy and informed decision-making. This aligns with person-centred care and suggests that communication behaviours observed in clinical role models may shape students’ understanding of rights-based and ethical practice.

Through this questioning, students embarked on a journey of self-discovery, ultimately understanding nursing beyond merely technical tasks (Theme 2). Students gained new awareness about their strengths, gender identity, and professional competencies. Similar to the study by Wang et al. [12], where first-year nursing students developed a deeper understanding of the multiple roles of nurses, our students transitioned from an initial task-oriented mindset to uncovering the true essence of the profession. They realized that technical interventions, while necessary, lose their significance without empathetic communication and respect. Hood et al. [44] and Choi et al. [45] also noted that participating in patient teaching and holistic care helps students identify crucial aspects of their professional identity. Furthermore, this realization aligns with the fact that nursing requires not only cognitive-academic skills but also essential personal qualities such as compassion, empathy, patience, and emotional awareness [46, 47]. This highlights that nursing education must transcend traditional academic metrics and actively foster these vital non-cognitive skills [12, 48].

The professional socialisation perspective also helps clarify why Theme 1 and Theme 2, although related, were treated as distinct analytical categories. Theme 1 reflects externally prompted professional identity questioning, as students evaluated the behaviours of practising nurses and articulated the kind of nurse they wished to become or avoid becoming. Theme 2, by contrast, reflects internally generated self-discovery arising from students’ own participation in care and their developing understanding of nursing beyond technical task performance. This interpretation is consistent with evidence showing that clinical supervisors, role modelling, ethical competence, preparation, self-confidence, reflective space and safety are central to nursing students’ professional identity development [27]. Recent evidence also indicates that the clinical learning environment is positively associated with nursing students’ professional identity, partly through learning engagement and stress-related growth [28]. Similarly, Steven et al. [49] highlighted emotional safety and tutoring as key conditions that help students process emotionally challenging clinical experiences. These findings support the interpretation that obstetric and gynaecologic placements may function as professional socialisation spaces where students negotiate values, observe role models, and develop a broader understanding of nursing beyond task completion. Our findings also resonate with recent international qualitative evidence from high-stress clinical settings. Hesami et al. reported that nursing students who witnessed patient cardiac arrest in the operating room experienced intense emotional reactions and identity-related struggles, with “Professional Identity Formation” emerging as one of the main themes [50]. Although conducted in a different clinical and cultural context, both studies suggest that emotionally demanding clinical encounters may contribute to professional identity development while highlighting the need for structured educational support.

Despite their strong desire to provide humanistic care, our students faced significant struggles and communication barriers when encountering emotionally challenging situations such as miscarriage, stillbirth, or adolescent pregnancy (Theme 3). They reported experiencing “emotional paralysis” and a sense of inadequacy. This finding strongly aligns with the literature indicating that obstetrics and gynaecologic units are emotionally demanding environments where perinatal losses are known to occur [51]. Studies have identified high levels of burnout and compassion fatigue among nurses in these settings [52–55]. Interestingly, a qualitative study by Mauri and Squillace [56] indicated that nurses in a termination of pregnancy unit mechanically performed their duties to shield themselves from emotional impact. This literature may help explain why our students might have observed the “task-focused” clinical nurses criticized in Theme 1; focusing on technical tasks may serve as a defence mechanism against emotional burden. It is important to note, however, that this explanation is inferential: the present study captured students’ perceptions of nurses’ behaviours rather than nurses’ own accounts of their motivations. The interpretation that task-focused behaviour reflects emotional self-protection is plausible and supported by the broader literature, but was not directly examined within this study. Future research employing mixed-methods designs that include the perspectives of both students and experienced nurses in obstetric and gynaecologic settings would be valuable in testing this interpretation. Therefore, providing structured psychological support and communication training may help both student and experienced nurses cope with these complex emotional barriers, although the effectiveness of such support strategies was not evaluated in the present study.

Students’ struggles with emotional support can also be interpreted in relation to emotional labour and compassion fatigue literature. Their accounts of being unable to speak, freezing, or hesitating when confronted with miscarriage, adolescent pregnancy, or task-focused interactions suggest that emotional support requires preparation for managing one’s own emotions while remaining present for patients. Recent studies show that recurrent pregnancy loss care involves heightened emotional labour and the need to balance empathy with professional boundaries [57], while compassion fatigue may occur during nursing internships and be shaped by resilience, empathy, and clinical placement context [58]. Chu [59] also reported that compassion fatigue may influence emotional labour in female nurses, particularly by increasing surface acting when emotional resources are depleted. These studies help contextualise why emotionally demanding obstetric and gynaecologic encounters may be difficult for students and why structured debriefing, emotional preparation, and supportive supervision may be needed. However, the interpretation that task-focused care may sometimes function as emotional self-protection remains literature-informed and inferential, because the present study captured students’ perceptions rather than nurses’ own motivations.

Finally, students described satisfaction with the care they offered and a sense of perceived self-efficacy when they perceived their actions as responsive to patients’ needs, particularly through education, problem-solving, and supportive presence (Theme 4). Consistent with our findings, previous studies have reported that nursing students may experience professional satisfaction and develop a more positive perception of nursing when they contribute meaningfully to patient care [17, 19, 60]. Although students described greater confidence and perceived competence in their narratives, these accounts represent subjective, retrospectively reported perceptions rather than objectively measured outcomes. Self-reported sentiments may be subject to recall bias, social desirability, or the emotional valence of the specific events selected for narration. The absence of complementary data sources—such as clinical instructor assessments, observational records, or validated self-efficacy scales (e.g., the Nursing Students’ Self-Efficacy Scale)—limits the interpretive claims that can be made about actual competence development. Future research could complement qualitative accounts with validated nursing self-efficacy instruments and clinical instructor assessments to further examine students’ perceived self-efficacy and competence development during obstetric and gynaecologic clinical practice.

These findings may also have implications for the transition from student learning to later professional practice. Although the present study focused on undergraduate nursing students rather than practising nurses, nurse-focused workforce research suggests that job crafting, leadership, dynamic capabilities, and innovative work behaviour may contribute to professional agency and adaptability [61, 62]. Alwali [62] reported that job crafting mediated the relationships between high-involvement work practices, servant leadership, and nurses’ innovative work behaviour, while another study showed that nurses’ dynamic capabilities were associated with innovative work behaviour and job performance [62]. These findings should be interpreted as broader workforce implications rather than direct explanations of the student narratives; they help position early clinical socialisation, reflective learning, and perceived competence as possible foundations for later adaptability, agency, and innovation in nursing work.

### Limitations

This study has several limitations. First, the study was conducted in a single nursing program and involved students undertaking one specific clinical placement; therefore, the findings should be interpreted as context-specific insights and may not be transferable to other educational or clinical contexts. Additionally, the participation of only two male students may not adequately reflect male students’ experiences in obstetric and gynaecologic settings. Male nursing students in these settings may encounter distinct challenges not captured in this dataset, including patient refusal of care, gendered role expectations from patients and staff, and feelings of marginalization within a predominantly female clinical environment. Such experiences may influence professional identity formation, confidence, integration into the care team, and long-term interest in the specialty in ways that differ from their female peers. Purposeful recruitment of male students in future research would enable a more comprehensive exploration of these gendered experiences.

Second, data were collected through a single written reflective narrative completed at the end of the clinical rotation. Although this approach enabled students to reflect on meaningful experiences, it may have been influenced by recall bias and a tendency to focus on particularly salient events. The use of a single data source also limited opportunities for methodological triangulation. Furthermore, claims related to self-efficacy were based solely on students’ self-reported perceptions and were not corroborated through additional sources, such as instructor evaluations or standardized self-efficacy measures. Future research could strengthen the depth and credibility of findings by combining reflective writing with in-depth individual interviews—which may be preferable to focus groups given the deeply personal and emotionally sensitive nature of many experiences described (e.g., perinatal loss, adolescent pregnancy, and intimate examinations)—as well as clinical observations or field notes collected throughout the placement period.

A further methodological consideration concerns the data collection prompt, which asked students to describe their “most impactful experience” during the placement. This framing likely privileged emotionally intense or narratively salient events over more routine, yet educationally significant, learning experiences. The reflective writing guide may also have shaped the types of experiences students selected and the way they narrated emotions, self-discovery and professional identity. Consequently, the findings may disproportionately represent exceptional encounters, while day-to-day learning processes, gradual skill development, and less dramatic clinical interactions may be underrepresented. Complementary approaches, such as diary-based reflection or episodic interviews conducted throughout the placement, may help capture a broader range of clinical learning experiences.

Finally, of the 135 eligible students, only 35 (26%) submitted reflective narratives. This relatively low participation rate raises the possibility of self-selection bias, as participants may have differed from non-participants in their motivation, engagement with clinical learning, comfort with self-disclosure, or willingness to reflect on their experiences. Students with particularly salient, emotionally meaningful, positive or negative experiences may have been more likely to participate, which may have contributed to a thematic structure that foregrounds emotional salience, professional identity questioning and humanistic care orientation. As a result, the findings may not fully represent the wider population of nursing students undertaking similar placements. In addition, only limited demographic information was collected. Including variables such as previous clinical experience, academic performance, and specialty interests in future studies would facilitate a more comprehensive assessment of variation across student experiences and enhance judgments regarding transferability.

## Conclusions

This study suggests that obstetric and gynaecologic clinical rotations can play an important role in shaping nursing students’ professional identity and emotional engagement with care. Using reflective writing, students described developing a stronger orientation toward humanistic, person-centred practice and a clearer view of nursing as more than technical task performance. They also reported difficulties providing emotional support in situations marked by vulnerability, loss, or distress, particularly when initiating communication or deciding what to say. At the same time, students described professional satisfaction and perceived self-efficacy when they perceived their actions as responsive to patients’ needs, particularly through practical problem-solving, patient education, and supportive presence.

These context-specific findings may inform nursing education and clinical practice. Clinical learning environments and the role modelling of practicing nurses may meaningfully influence students’ professional socialization. Communication skills training, supervised reflective learning, and structured guidance for emotionally challenging encounters may be useful educational strategies to consider, although their effectiveness was not tested in this study. Positive, humanistic role modelling in clinical settings may also support students’ perceived confidence and their orientation towards responding to women’s holistic care needs.

## Supplementary Information

Below is the link to the electronic supplementary material.

Supplementary Material 1

## Data Availability

The datasets are not publicly available due to the sensitive nature of reflective narratives but are available from the corresponding author on reasonable request, subject to ethical approval/permissions.

## Abbreviations

SRQR: Standards for Reporting Qualitative Research
