# Supplementary material for: “What Kind of Nurse Will I Be?”: a qualitative descriptive study of nursing students’ experiences in obstetric and gynaecologic clinical practice
Source: BMC Nurs. 2026 Aug 8;25:1067. doi: 10.1186/s12912-026-05160-3 (PMC13732973; doi:10.1186/s12912-026-05160-3)
Supplement: Supplementary file 1 [file 12912_2026_5160_MOESM1_ESM.pdf]

REFLECTIVE CARE STORY WRITING GUIDE

Research Topic: Nursing Students' Care Experiences in Obstetric and Gynecologic Clinical Practice

Hello,

The purpose of this study is to gain an in-depth understanding of the care experiences of nursing students participating in the obstetrics and gynecology clinical practice for the first time. We kindly ask you to write a care story reflecting the situations you encountered, the emotions you experienced, and your professional discoveries while providing care in the clinical setting. There are no right or wrong answers in this story; what matters most is your authentic experience. We would like you to know that everything you write is highly valuable to us and will make significant contributions to nursing education.

The information you provide will be used solely for scientific purposes. Your personal information will be kept strictly confidential when reporting the research results, and the data will be evaluated using codes/pseudonyms. Your participation in this research is entirely voluntary. We assure you that whether or not you participate, and the content of what you write, will have absolutely no impact on your clinical practice grades or performance evaluations. We thank you for participating in this research and sharing your experiences with us.

Please deeply reflect on your obstetrics and gynecology clinical practice. Using the guiding questions below as a framework, please write a continuous and fluent care story that covers all these areas, rather than answering the questions item by item:

1. *Specific Care Experience:* Describe a real-life patient care story or situation that you witnessed and actively participated in during your clinical practice, which impacted you the most. What exactly happened?

2. Internal Emotional and Cognitive Responses: What did you feel, and what were your thoughts regarding this specific situation or patient?

3. *Clinical Observations and Nursing Actions:* What clinical observations did you make, and what nursing care did you provide regarding this specific situation or patient? (If there was a situation where you could not intervene, what would you have liked to do or how would you have liked to act?)

4. *Overall Impact:* How did this specific experience affect you emotionally and in terms of your perspective regarding patient care? What were the aspects that impacted you the most?

5. *Professional Self-Discovery:* What did you discover about yourself, your professional identity, or your personal care philosophy while providing care in this specific situation?
